# Supplementary figures and images for: Ecological associations of the coastal marsh periwinkle snail Littoraria irrorata: field and laboratory evidence of vegetation habitat preferences
Source: PeerJ. 2025 Mar 12;13:e19071. doi: 10.7717/peerj.19071 (PMC11910147; doi:10.7717/peerj.19071)

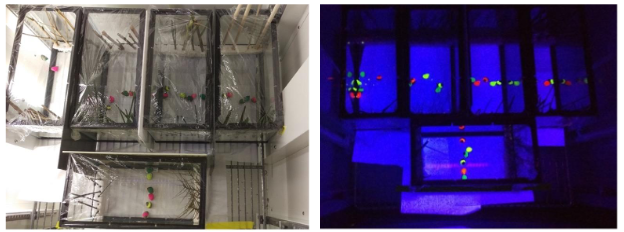

Supplement: Supplemental Information 1 — Sets of S. alterniflora, S. patens, D. spicata, J. roemerianus and wooden dowels were mounted on opposite sides using Styrofoam inserts, and six snails were placed in the center of each arena. Cameras were mounted above to record snail movement. [file peerj-13-19071-s001.png]

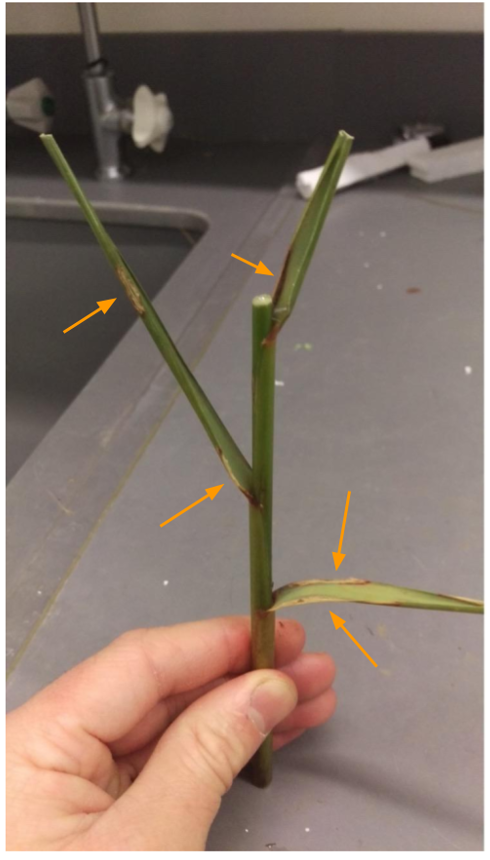

Supplement: Supplemental Information 2 — Radulations were concentrated on leaves of S. alterniflora plants, and were observed on many of the plant segments used in experimentation. [file peerj-13-19071-s002.png]
